# High-resolution structure of a type IV pilin from the metal-reducing bacterium *Shewanella oneidensis*

**DOI:** 10.1186/s12900-015-0031-7

**Published:** 2015-02-27

**Authors:** Manuela Gorgel, Jakob Jensen Ulstrup, Andreas Bøggild, Nykola C Jones, Søren V Hoffmann, Poul Nissen, Thomas Boesen

**Affiliations:** Department of Molecular Biology and Genetics, Aarhus University, Gustav Wieds Vej 10c, Aarhus C, 8000 Denmark; ISA, Department of Physics and Astronomy, Aarhus University, Ny Munkegade 120, building 1525, Aarhus C, 8000 Denmark

**Keywords:** Type IV pili, Nanowire, PilBac1, PilA, *Shewanella oneidensis*, X-Ray Crystallography, SAXS, SRCD

## Abstract

**Background:**

Type IV pili are widely expressed among Gram-negative bacteria, where they are involved in biofilm formation, serve in the transfer of DNA, motility and in the bacterial attachment to various surfaces. Type IV pili in *Shewanella oneidensis* are also supposed to play an important role in extracellular electron transfer by the attachment to sediments containing electron acceptors and potentially forming conductive nanowires.

**Results:**

The potential nanowire type IV pilin Pil_Bac1_ from *S. oneidensis* was characterized by a combination of complementary structural methods and the atomic structure was determined at a resolution of 1.67 Å by X-ray crystallography. Pil_Bac1_ consists of one long N-terminal α-helix packed against four antiparallel β-strands, thus revealing the core fold of type IV pilins. In the crystal, Pil_Bac1_ forms a parallel dimer with a sodium ion bound to one of the monomers. Interestingly, our Pil_Bac1_ crystal structure reveals two unusual features compared to other type IVa pilins: an unusual position of the disulfide bridge and a straight α-helical section, which usually exhibits a pronounced kink. This straight helix leads to a distinct packing in a filament model of Pil_Bac1_ based on an EM model of a *Neisseria* pilus.

**Conclusions:**

In this study we have described the first structure of a pilin from *Shewanella oneidensis*. The structure possesses features of the common type IV pilin core, but also exhibits significant variations in the α-helical part and the D-region.

**Electronic supplementary material:**

The online version of this article (doi:10.1186/s12900-015-0031-7) contains supplementary material, which is available to authorized users.

## Background

Type IV pili are found in many Gram-negative bacteria as well as in some Gram-positive bacteria and archaea, where they function in numerous cellular processes including adhesion, DNA transfer and virulence [1-6].

Furthermore, in the metal reducing bacteria *Shewanella oneidensis* and *Geobacter sulfurreducens* type IV pili have been implicated in extracellular electron transport (EET) pathways [7-9]. Both of these organisms can respire on a variety of electron acceptors, including metals such as iron, manganese and uranium oxides, which has made these organisms attractive research targets in the fields of environmental sciences and nanotechnology [10-15]. *S. oneidensis* and *G. sulfurreducens* can reduce extracellular electron acceptors directly through membrane bound cytochromes [16-19]; *S. oneidensis* can also produce soluble electron shuttles to transfer electrons to extracellular acceptors [20-22]. To allow for highly efficient electron transfer rates, *S. oneidensis* and *G. sulfurreducens* can form biofilms in which strong cell-cell interactions and contact between cells and insoluble electron acceptors are beneficial in certain habitats [23-25]. Such an attachment function is expected to implicate type IV pili.

Type IV pili have been associated with a more direct role in EET. Both *S. oneidensis* and *G. sulfurreducens* can form conductive filaments that transfer electrons extracellularly over multiple cell lengths from one cell to another and from a cell to an electron acceptor [7,9]. These filaments were collectively termed nanowires. While it was clearly shown that nanowires in *G. sulfurreducens* were made of the type IV pilin PilA, the exact subunits of nanowires in *Shewanella* have not been identified so far. Yet, there has been strong evidence that nanowires are made of proteins and studies have indicated the contribution of pili in extracellular electron transport [9,26] – whether this is due to an indirect role by attaching to electron acceptors or due to a direct role by nanowire formation, is not clear at this point. Altogether, the high overall similarity between *G. sulfurreducens* and *S. oneidensis*, including metabolic pathways, the prevalence of multiheme cytochromes and the formation of conductive filaments, makes it very likely that nanowires in both species are formed in a similar way and function based on the same principles.

Currently, two major hypotheses prevail on how nanowires transfer electrons. The metallic-like conductivity theory claims that type IV pili are the conductive units themselves [27]. The aromatic amino acids in PilA are supposedly aligned so closely that the π-electrons can be delocalized and be transferred along the pilus like in a metal lattice. According to the alternative multi-step hopping theory, type IV pili only form the backbone of nanowires to which multiheme cytochromes such as MtrC and OmcA in *S. oneidensis* and OmcS and OmcZ in *G. sulfurreducens*, respectively, attach [28,29]. The electrons can then hop from one heme of one protein to another heme of the neighboring protein. So far, the electron transfer mechanism along bacterial nanowires is not clear yet and the discussion, on which of the two mechanisms is true, is ongoing [27,28,30,31].

Type IV pilins (T4Ps) build up the polymeric pilus in a repetitive way [32-34]. Two kinds of type IV pilins have been described, type IVa and IVb pilins (T4aPs and T4bPs, respectively). These two types are primarily distinguished by the length of their leader sequences with T4aPs containing an N-terminal leader sequence of 6 to 7 residues, whereas the leader sequences in T4bPs range from 15 to 30 [3]. Generally, T4aPs are synthesized as pre-pilins in the cytoplasm and are guided to the inner membrane by their N-terminal leader sequence [35], which is then cleaved off at a conserved cleavage site by the leader peptidase PilD at the cytoplasmic face of the inner membrane [36,37]. The new N-terminus (commonly a phenylalanine) is then methylated and the processed pilins are inserted into the pilus by an inner membrane multimeric complex (including the assembly ATPase PilB) [38-40] and the assembled pilus is fully exported into the extracellular space via the outer membrane secretin PilQ [41,42]. The N-terminal transmembrane domain of type IV pilins is an approximately 20 residues long hydrophobic α-helix, which has a highly conserved sequence among different species. Downstream of this sequence motif, the sequence variability of T4aPs is however very high and the total length of pilin proteins can vary from 90 residues (*G. sulfurreducens*) [43] to more than 150 residues (*P. aeruginosa*) [44].

T4aPs share the signature of the N-terminal leader sequence and the transmembrane α-helix with pseudopilins [45] Like a type IV pilus, a pseudopilus extends from the inner membrane into the periplasm, but it does not go beyond the outer membrane [46] (reviewed in [47,48]). Instead, it is associated with the type II secretion system and is involved in the secretion of virulence factors from the periplasm to the extracellular environment [49].

So far, more than 10 structures of different T4Ps and more than 9 structures of pseudopilins have been deposited in the protein data bank (Additional file 1: Table S1). However, only four structures of a full‐length T4P are available. All other T4P structures – and all pseudopilin structures – are of N‐terminally truncated constructs that do not include the N‐terminal hydrophobic transmembrane α‐helix. This is unfortunate, as this part is the most conserved part among T4Ps and pseudopilins. Still, all structures of T4aPs and pseudopilins exhibit a conserved central core of a long N-terminal α-helix packed against three to four antiparallel β-strands [32,50-52]. However, various structural elements can be inserted around this conserved core allowing for the diverse functions of T4Ps. In 2013 the structure of the nanowire associated pilin PilA from *G. sulfurreducens* was determined by NMR spectroscopy revealing a single, 61 residue long α-helix [43], but as yet, no structure of a T4P from *S. oneidensis* is available.

In this work, we have determined the structure of the putative nanowire associated T4P on the gene locus SO_0854 [Uniprot: q8eii5] from *S. oneidensis* by X-Ray crystallography to a resolution of 1.67 Å. This T4P from *S. oneidensis* shares the highest degree of sequence identity to PilA from *G. sulfurreducens* (48%) when comparing the first 61 residues after the cleavage site (which corresponds to the full length of PilA from *G. sulfurreducens*). (Additional file 2: Table S2, Additional file 3: Figure S1). The structure reveals the conserved fold of a type IV pilin with a parallel dimer in the asymmetric unit. We have also used Small Angle X-Ray Scattering (SAXS) and Synchrotron Radiation Circular Dichroism (SRCD) to characterize the structure and stability of this protein in solution.

## Results and discussion

### Sequence conservation and position in the genome

**Figure 1:**
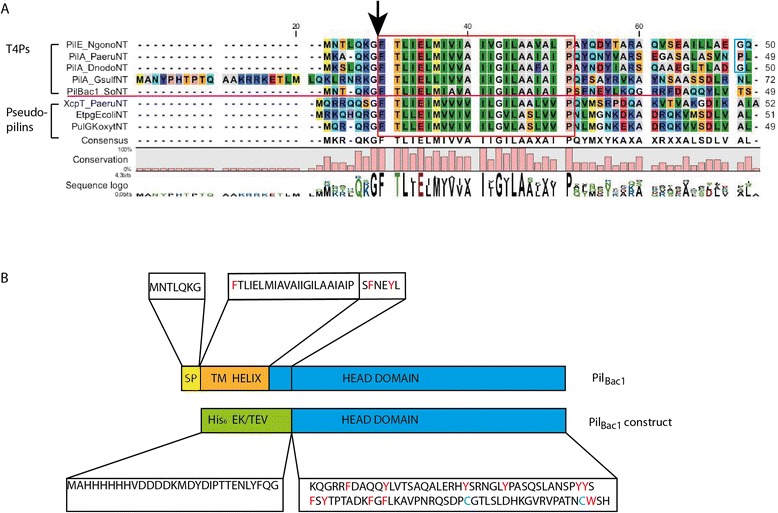
**Sequence alignment and construct of Pil**_**Bac1**_ **. A**: Sequence alignment of the N-terminal part of Pil_Bac1_ (Pil_Bac1_NT) with the N-terminal parts of type pilins and pseudopilins. Type IV pilins: PilE from *N. gonorrhoeae*, PilA from *P. aeruginosa*; PilA from *D. nodosus,* PilA from *G. sulfurreducens.*; Pseudopilins: XcpT from *P. aeruginosa*; EpsG from *V. cholerae*; EtpG from *E. coli*; PulG from *K. oxytoca*. The protease cleavage site is marked with an arrow and the hydrophobic transmembrane helix is framed in red and the residues introducing kink2 in blue. The alignment was performed with the program MUSCLE installed in CLC Genomics Workbench 6.9.1 [53]. **B**: Construct of Pil_Bac1_∆N. SP: leader sequence; TM HELIX: transmembrane α-helix; His_6_: 6 residue long Histidine tag; EK: enterokinase cleavage site; TEV: TEV protease cleavage site. The aromatics and the cysteines are highlighted in red and blue in the sequence respectively.

The pilin protein encoded by the gene locus SO_0854 exhibits the conserved N-terminal leader sequence (MNTLQKG) and a hydrophobic patch of 22 residues expected to form a transmembrane helix, which is the hallmark of both type IV pilins and pseudopilins [45] (Figure 1A). Additionally, it possesses two conserved cysteines in the C-terminal part (Cys96, Cys113) which form a disulfide bridge in T4Ps. Based on these features, we classified the protein SO_0854 as a type IV pilin and added it to the subclass of T4aPs due to its short leader sequence.

So far, no consistent nomenclature for T4Ps has been established and therefore, newly described T4Ps cannot be named unambiguously. The low sequence similarity among T4Ps further complicates the naming process. For this reason, the naming of the T4P on the gene locus SO_0854 will briefly be outlined here. SO_0854 is the first open reading frame in a gene cluster consisting of three other putative type IV pilins (SO_0853, SO_0852, SO_0851) and a type IV pilin adhesin with a bactofilin motif (SO_0850). Bactofilins are fiber forming, membrane attached proteins that have been identified in many Gram-negative bacteria and they are associated with cytoskeleton related functions such as cell motility, cell morphology and cell division [54,55]. In *M. xanthus* the polymerized bactofilin BacP directly interacts with PilB and PilT which are responsible for extension and retraction of type IV pili, respectively, and thus for the motility of the cell [56]. In *S. oneidensis* a bactofilin (SO_1662) [53] was shown to localize to the cell division ring and this bactofilin was therefore assumed to be associated with cell division [54]. Even though bactofilins constitute a recently discovered protein family and their functions have not been fully elucidated yet, the finding of this motif in the putative adhesin in this operon is intriguing. For this reason, we named the five pilin proteins on the gene loci SO_0854, SO_0853, SO_0852, SO_0851 and SO_0850 Pil_Bac1_, Pil_Bac2_, Pil_Bac3_, Pil_Bac4_ and Pil_Bac5_ respectively.

### Construction and purification of a soluble construct

To obtain a soluble version of Pil_Bac1_, a construct was designed that lacks the N-terminal 35 residues including the signal peptide and the transmembrane α-helix. Instead, a His-tag and a TEV protease cleavage site were inserted to enable tag removal (leaving one N-terminal glycine) during the purification process (Figure 1B). This construct was termed Pil_Bac1_ΔN. The protein was well-expressed in *E. coli* and could be purified to homogeneity in a two-step purification procedure using two passes over a Ni-column (before and after tag removal) followed by size exclusion chromatography. Size exclusion chromatography of Pil_Bac1_ΔN gave a monodisperse peak and, comparing the elution volume with those of globular standard proteins that were used for calibration of the size exclusion column, a molecular weight of 11 kDa was estimated, which is close to the theoretical monomeric weight of 9.9 kDa.

### Thermostability of Pil_Bac1_ΔN

**Figure 2:**
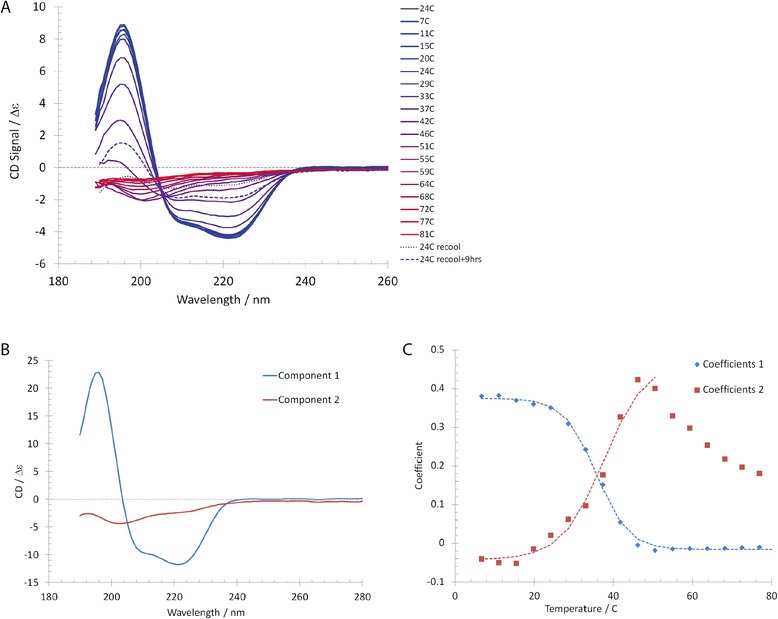
**Temperature scan of Pil**_**Bac1**_ **ΔN using SRCD. A**: SRCD spectra with increasing temperature shown with data from 260 to 190 nm. **B**: Plot of the component curves resulting from principal component analysis of the CD data set in A, with the associated coefficients plotted in C. **C**: A fit of the coefficients for component 1 (blue) with a sigmoidal function to find the inflection point yields a melting temperature of 36°C, while fitting up to 50°C for component 2 yields a value of 38°C.

The stability of Pil_Bac1_ΔN was assessed by SRCD measurements where the temperature was increased stepwise from 7 to 81°C and data were recorded from 280 nm to 190 nm (Figure 2). With increasing temperature the signal strength at 195, 210 and 222 nm fell, indicating a change or a loss of structure. Interestingly, this effect was partly reversed when re-cooling the sample back to 24°C indicating that the protein could, at least partially, refold. The change of the structure was analyzed with principle component analysis (PCA) (Figure 2B) and the contribution of the different components relative to the temperature is shown in Figure 2C. The inflection points of both curves yield an approximate melting temperature of 36 and 38°C respectively. A somewhat higher value (42°C) was obtained in a Thermofluor assay (data not shown). In the Thermofluor experiment, a steeper gradient was applied and this might have resulted in a higher melting temperature compared to the SRCD measurements. In general, a melting temperature around or below 40°C has been claimed to counteract crystallization [57].

### X-ray crystallography

An initial hit was obtained in the Structure Screen from Molecular Dimensions in condition 42 (0.2 M (NH_4_)_2_SO_4_, 30% PEG 8,000) at 15 mg/ml at 19°C. These crystals could not be reproduced when manually recreating the conditions. Introducing 100 mM CHES pH 8.6 as a buffer component into the crystallization condition yielded crystals of around 50x50x500 μm^3^. Additionally, the ratio between protein and reservoir solution was increased from 1:1 to 2:1. The best looking crystals were consistently obtained at 26% PEG 8,000, 0.15 M (NH_4_)_2_SO_4_ and 0.1 M CHES pH 8.6. The crystal structure of Pil_Bac1_ΔN was initially determined by sulfur SAD at 2 Å resolution based on the anomalous signal of the two cysteines in the C-terminal domain, a bound sulfate molecule and a bound sodium ion (structure determination described in another manuscript). A high resolution data set was collected on another crystal and data were processed in the high symmetry space group I222 to a resolution of 1.67 Å using the CC_1/2_ value as a cut-off [58] (Table 1). The model obtained from S-SAD was then used as a search model in molecular replacement for this data set, and all residues were modelled into clear electron density with no apparent ambiguities. The crystal form had a large solvent content (62%) yielding a high optical resolution and favorable data-to-parameter ratio at 1.67 Å resolution. Riding hydrogen atoms were included in the last model refinement and this resulted in a decrease in R_*free*_ of 1%.

**Table 1:** **Data collection and processing statistics for the structure of Pil**_**Bac1**_ **ΔN**

**Subset**	**Native**
Crystallization condition	26% PEG 8,000, 0.1 M CHES pH 8.6, 0.15 M (NH_4_)_2_SO_4_
Beamline	BL-14.2, BESSY-II, Helmholtz Zentrum Berlin, DE
Detector	Rayonix MX225
Wavelength (Å)	0.918409
Crystal to Detector Distance (mm)	150.0
Rotation/ Frame (°)	0.5
Number of Frames	200
*Data Processing statistics*	
Resolution (Å)	48.23-1.67 (1.70-1.67)
Space group	I 2 2 2
Unit cell parameters (Å, °)	48.91, 96.46, 110.33; 90.0, 90.0, 90.0
No. of unique reflections	56,946 (4,148)
No. of total reflections	111,644 (8,093)
Multiplicity	1.96 (1.95)
Completeness (%)	97.0 (95.1)
*R*_merge_^a^	0.039 (0.641)
*R*_r.i.m._^b^	0.054 (0.890)
Wilson B-factor (Å^2^)	23.12
Mean *I*/*σI*	13.3 (1.2)
CC_1/2_	0.999 (0.578)
*Refinement statistics*	
*R*_work_	0.1798
*R*_free_	0.2075
Number of non-hydrogen atoms modelled	1631
Number of non-hydrogen protein atoms	1406
Number of ligand atoms	6
Number of solvent molecules	219
R.M.S.D. from ideal values	
Bonds (Å)	0.004
Angles (°)	1.101
Ramachandran	
Favoured (%)	99
Outliers (%)	0
Clash Score	0.73
Average B-factor (Å^2^)	34.6
Average B-factor for protein (Å^2^)	33.7
Average B-factor for ligands (Å^2^)	61.4
Average B-factor for solvent molecules (Å^2^)	39.7

Data were processed with XDS [59]. The structure was determined and built and refined with Phenix and COOT [60,61]. Values in parentheses are given for the highest-resolution shell.

^a^:*R*_merge_ = $$ {\displaystyle \sum_{hkl}{\displaystyle \sum_i\left|Ii(hkl)-\overline{I(hkl)}\right|}}{\displaystyle \sum_{hkl}{\displaystyle \sum_iIi(hkl)}} $$ [62]; ^b^: *R*_.r.i.m_. = $$ {\displaystyle \sum_{hkl}{\left[N/\left(N-1\right)\right]}^{{\scriptscriptstyle \frac{1}{2}}}}{\displaystyle \sum_i\Big|}Ii(hkl)-\overline{I(hkl)}\Big|{\displaystyle \sum_{hkl}{\displaystyle \sum_iIi(hkl)}} $$ [62].

Additional density was observed at the interface to the large solvent channels of the crystal. This density could not be attributed to solvent or an additional copy of the protein, but probably integrates features of partially associated molecules from the mother liquor or buffer solutions used for purification, such as PEG, glycerol, ions and water (Additional file 4: Figure S2).

### Overall structure

**Figure 3:**
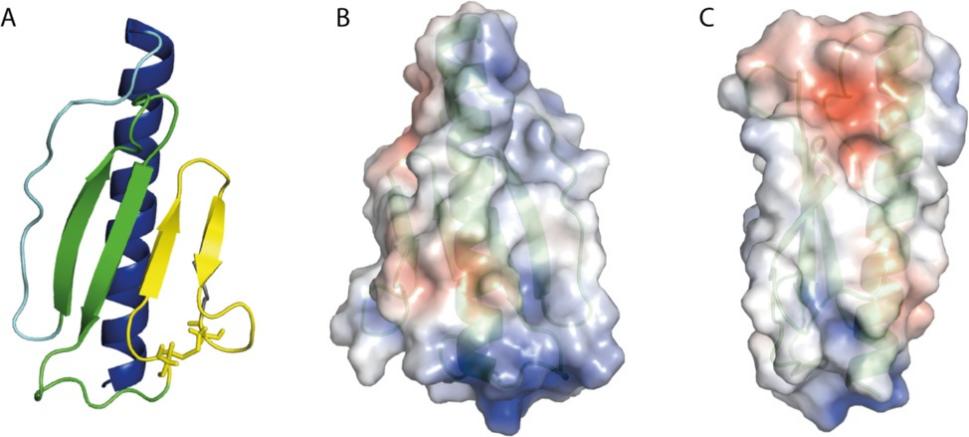
**Overall structure of Pil**_**Bac1**_ **ΔN. A**: Pil_Bac1_ contains a long N-terminal α-helix (blue), an unstructured αβ-loop (light blue), 4 antiparallel β-strands (green and yellow) and a conserved D-region framed by the two cysteines forming a disulfide bridge (yellow). The oxidized cysteines are shown as sticks. **B, C**: Electrostatic potential of Pil_Bac1_∆N. Red: negatively charged; blue: positively charged.

Pil_Bac1_ΔN consists of one long N-terminal α-helix packed against 4 antiparallel β-strands resembling the core fold of type IV pilins (Figure 3A). A long loop forms the αβ-loop connecting the α-helix and the first β-strand. This region is among the most variable parts in T4P structures and can contain insertions of a short α-helix or a short β-strand or simply display a random coil loop structure as it is the case for Pil_Bac1_ΔN. The two cysteines in the loops b2-b3 and the loop after b4 indeed form the conserved disulfide bridge of T4Ps that forms the disulfide-bounded loop region (D-region) and keeps strands 3 and 4 together.

Pil_Bac1_ΔN exhibits two positively charged surface patches at the N- and at the C-terminus of the α-helix due to closely spaced arginines and lysines facing the solvent area (Figure 3B and C). A negatively charged patch is formed by residues in the C-terminus and the top of the head domain. These charged regions might act as a platform for interactions with other molecules, for example with other pilins in the pilus or receptors for attachment. For instance, in the structure of PilE from *Neisseria gonorrhoeae* docked into an EM envelope of a pilus, positively charged patches were suggested to be responsible for DNA binding [34]. For a pilin protein from *S. oneidensis* such as Pil_Bac1_, potential binding partners could be multiheme cytochromes which were suggested to be the electron transporting components in nanowires [28,29]. In *G. sulfurreducens*, the multiheme cytochrome OmcS was shown to co-localize with nanowires suggesting a direct interaction [63] and a similar interaction can be expected from multiheme cytochromes in *S. oneidensis*.

### Comparison to other type IV pilins and pseudopilins

Pil_Bac1_ΔN is structurally similar to other T4Ps in the α-helix and the first two β-strands. However, variable regions are also characteristic of type IV pilins. These include the αβ-loop and the loops connecting the β-strands and parts of the D-region. The loops between the β-strands have been proposed to be involved in contact formation with interaction partners [34]. In Pil_Bac1_ΔN, loops b1-b2 and b2-b3 are relatively long. Compared to other type IV pilins, Pil_Bac1_ΔN is very compact without any additional motifs or insertions, mostly due to its short sequence relative to other T4P head domains. In the reported structures of T4aPs, a disulfide bridge is usually formed between cysteines in the fourth β-strand (b4) and the last loop (Additional file 1: Table S1). In contrast, in Pil_Bac1_ the first cysteine is not situated in b4, but in the loop from b2 to b3. Another exception is the structure of PilA_4 in which the disulfide bridge is formed by two cysteines in β-strands b3 and b4 [64].

In general, structures of full-length T4Ps exhibit two kinks in the long N-terminal α-helix, one in the transmembrane part (kink 1) and one in the C-terminal part of the α-helix (kink 2) (Additional file 1: Table S1). These two kinks are due to helix breaking residues (prolines or glycines) at positions 22 and 42, which are conserved among most T4aPs. Interestingly, Pil_Bac1_ possesses a helix breaking proline at position 22, but a helix breaking residue is missing at position 42 (Figure 1). Therefore, kink 2 is missing in Pil_Bac1_ and the α-helix in the structure of Pil_Bac1_ΔN is straight. Such a feature has commonly been observed in structures of pseudopilins and in the T4**b**Ps PilS from *S. typhi* and TcpA from *V. cholerae* as well as in two T4a pilins, namely PilA from *G. sulfurreducens* and PilA_4 from *T. thermophilus.* The feature of a straight α-helix could suggest a different mode of packing in the pilus (see below).

**Figure 4:**
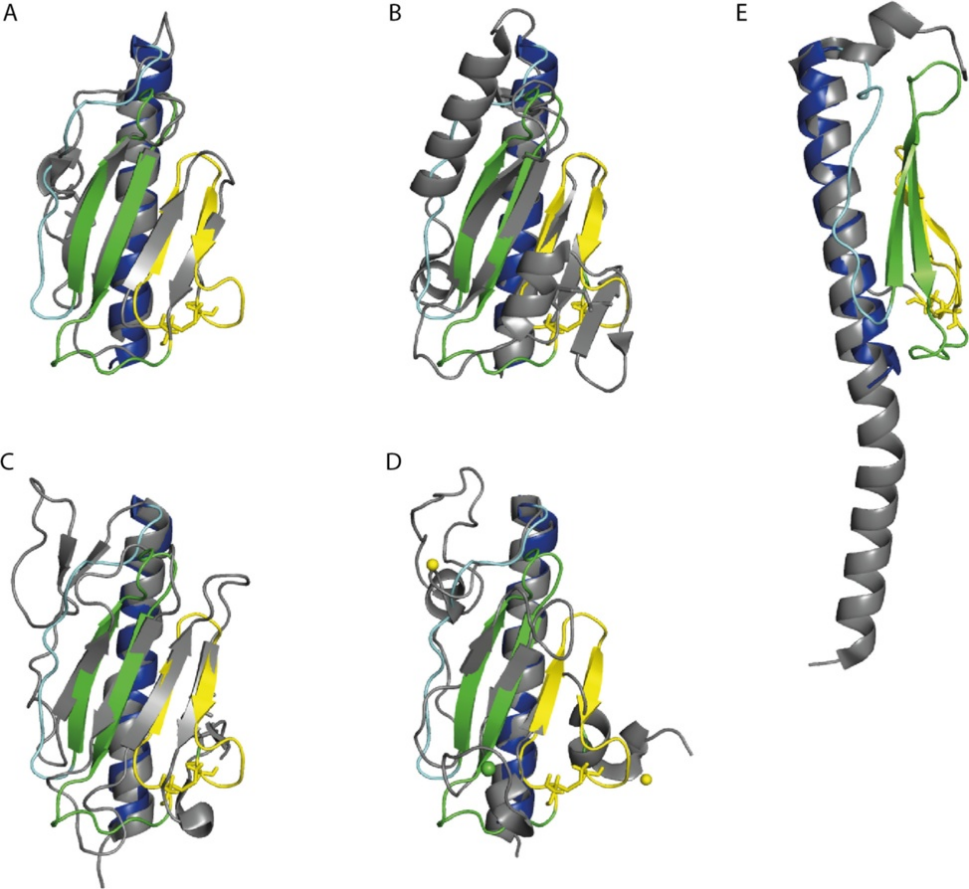
**Overlay of the structure of Pil**_**Bac1**_ **ΔN with T4Ps and a pseudopilins. A**: PilA_4 from *T. thermophilus* (4BHR.PDB), **B**: PilX from *N. meningitides* (2OPD.PDB), **C**: the PAK pilin from *P. aeruginosa* (1X6Z.PDB), **D**: the pseudopilin PulG from enterohaemorraghic *E. coli* (4LW9.PDB) and **E**: PilA from *G. sulfurreducens*. Pil_Bac1_∆N is coloured as in Figure 3A. The overlaid structure is shown in grey. A calcium ion and two zinc ions in D are shown in green and yellow, respectively.

To identify homologous structures to Pil_Bac1_ΔN, a search with the DALI server [65] was performed (Additional file 5: Table S3). As expected, structures of type IV pilins scored the highest and among them, the highest score was seen with the T4aPs PilA_4 from *T. thermophilus* [64] (4BHR.PDB; DALI: Score: 8.7), followed by the minor pilin PilX from *N. meningitis* [66] (2OPD.PDB; DALI: Score: 8.3) and the PAK pilin from *P. aeruginosa* [67] (1X6Z.PDB; DALI: Score: 8.1) (Figure 4A-C). PilA_4 is one of the exceptions of T4aPs with a straight, continuous α-helix and superimposes well with Pil_Bac1_ΔN (Figure 4A). The biggest variation between PilA_4 and Pil_Bac1_ΔN lies in the αβ-loop, which forms a short α-helix in PilA_4 and a random coil structure in Pil_Bac1_ΔN.

Pil_Bac1_ΔN also shares structural similarity with pseudopilins, which superimpose well in the core regions (Figure 4D). As for other type IV pilins, the biggest differences are observed in the variable regions. Additionally, pseudopilins coordinate a calcium ion in the D-region, where Pil_Bac1_ΔN and other type IV pilins form a conserved disulfide bridge [68].

Pil_Bac1_ΔN overlays well with the α-helix of PilA from *G. sulfurreducens* and the feature of a straight α-helix after the transmembrane part (Figure 4E); yet, this T4aP structure was not scored high by the DALI server, as PilA from *G. sulfurreducens* does not contain a head domain. Noteworthy, a gene (GSU1497) is located directly downstream of pilA that codes for a protein that was shown to be up‐regulated together with PilA and a few multiheme cytochromes in microbial fuel cells [69]. Furthermore, PilA was not detected by western blotting in strains deficient for GSU1497 [70]. Therefore, this protein might constitute the missing head domain of PilA from *G. sulfurreducens* and a future structural comparison of this protein with Pil_Bac1_ΔN would be very interesting.

### Dimeric interface

**Figure 5:**
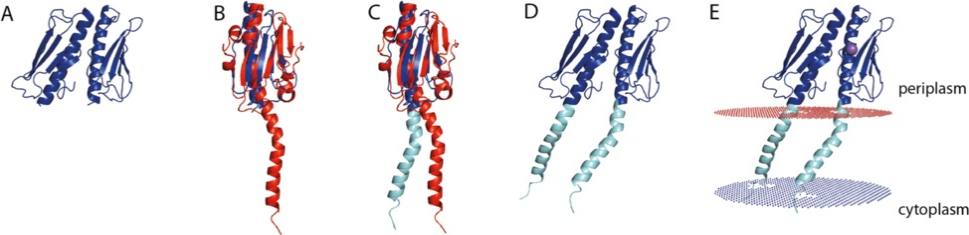
**Dimer of Pil**_**Bac1**_ **ΔN. A**: Dimeric interface between two Pil_Bac1_∆N molecules in the crystal. **B**: Superposition of a Pil_Bac1_∆N monomer with PilE from *N. gonorrhoeae* (2HI2.PDB). **C**: Superposition based on the head domains of a Pil_Bac1_∆N monomer with the modelled α-helix onto PilE from *N. gonorrhoeae* (2HI2.PDB). **D**: Pil_Bac1_∆N dimer with the modelled α-helices at the N-terminus based on a superposition with the α-helix from PilE from *N. gonorrhoeae* (2HI2.PDB). **E**: Potential arrangement of a Pil_Bac1_ dimer in a membrane. This figure was generated with the PPM server [79]. The structure of Pil_Bac1_∆N is shown in blue, the modelled helix in cyan and PilE from *N. gonorrhoeae* is shown in red.

Pil_Bac1_ΔN was crystallized as a parallel dimer in the asymmetric unit in which the interface is formed by interactions between 15 and 20 residues in the α-helix from monomers A and B, respectively (forming a non-proper dimer with a screw-axis) (R.M.S.D. of 0.309 Å based on 79 out of a total of 89 C_α_s)(Figure 5A). Previously it was noted that pilins can exist as dimers and multimers [71-73]. Many structures of type IV pilins and pseudopilins were also determined in a dimeric [32,68,74-78] or even in a trimeric state [68]. However, different to Pil_Bac1_ΔN, most of them were not arranged in a physiologically relevant conformation (e.g. antiparallel, in-line). The structures of the T4P CofA from *E. coli* (3S0T.PDB) and of the pseudopilin PulG from *E. coli* (3G20.PDB) were determined as dimers, in which the monomers are further apart from each other. This dimerization was probably caused by crystal contacts. In contrast, in the structure of full-length FimA from *D. nodosus*, the two monomers are held together by extensive, intermolecular interactions. However, unlike Pil_Bac1_ΔN, the dimeric interface is here formed between the transmembrane domains.

Since the N-terminal transmembrane domain in Pil_Bac1_ΔN is missing in the structure, we modelled this conserved part based on the full-length structure of mature PilE from *N. gonorrhoeae*, as this region is highly conserved in T4Ps as a transmembrane α-helix (Figures 1 and 5A–D; R.M.S.D. of the α-helices and the first two β-strands: 1.7 Å). This additional domain extends the α-helices in both monomers in the crystal dimer by a further 28 residues without the introduction of any clashes (Figure 5E). Consequently, this arrangement maintains contacts between both monomers and allows for the existence of the dimer in a membrane (Figure 5E). In the modelled part of the alpha helix, a kink is introduced due to the presence of a conserved helix breaking proline in the transmembrane helix at position 22 (kink1). Such a kink has been described in all four available full-length structures of T4Ps [32,43,77,80] and separates the N- and the C-terminal parts of the helix from each other.

**Figure 6:**
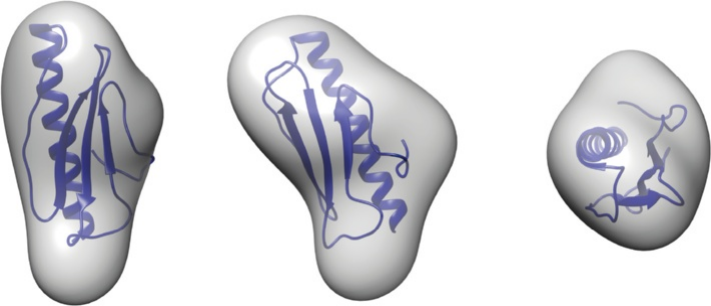
**Crystal structure of Pil**_**Bac1**_ **ΔN docked into its SAXS envelope.** A dummy atom model from the SAXS data was generated with DAMMIF [81] and superposed onto the crystal structure with SUPCOMB [82]. This oriented SAXS model was then converted into an envelope with the pdb2vol software from the SITUS package [81,85].

To assess the oligomeric state of Pil_Bac1_ΔN in solution we performed SAXS studies at concentrations ranging from 1 – 16 mg/ml (Additional files 6: Table S4, Additional file 7: Figure S3). No signs of aggregation or repulsive forces were observed from the scattering data at any of these concentrations as judged by comparison of the scattering intensities at low scattering angles for all concentrations used. Guinier and Porod analysis revealed a radius of gyration of 14 and 16 Å respectively, and the molecular mass determined by Porod volume analysis indicated a molecular weight of only 6 kDa, which is below the theoretical molecular mass of 9.9 kDa. Kratky analysis indicated a well-folded molecule (Additional file 7: Figure S3). Altogether, these findings indicated that Pil_Bac1_ΔN existed as a monomer in solution. A dummy atom (DA) model of Pil_Bac1_ΔN was built by the program DAMMIF based on the SAXS data [81] and the crystal structure of monomeric Pil_Bac1_ΔN compared to the DA model using the program SUPCOMB [82], yielding a good fit. In UCSF Chimera [83] a simulated map at 10 Å based on the crystal structure was fitted to a SAXS envelope based on the DA model giving a CC of 0.762 (Figure 6). A scattering curve was calculated on the basis of the crystal structure using the program CRYSOL [84] and compared to the experimentally measured scattering data. This resulted in a chi value (discrepancy between the theoretical and experimental scattering curve) of 1.82, which is indicative of good agreement.

The results from the SAXS analysis were further substantiated by size exclusion chromatography and a PISA analysis [86], which indicated that the Pil_Bac1_ΔN dimer interface energy was low. However, the environment in a membrane with potentially interacting transmembrane helices is very different from that of a truncated protein in solution, the local concentration of pilins is significantly higher and the degree of translational freedom is reduced in a membrane. Therefore we cannot exclude a possible function of dimeric Pil_Bac1_ in the membrane, but further studies on the full-length Pil_Bac1_ in membranes and assembled into pili will be needed to evaluate oligomeric states of Pil_Bac1_.

### Na^+^-Ion binding site

**Figure 7:**
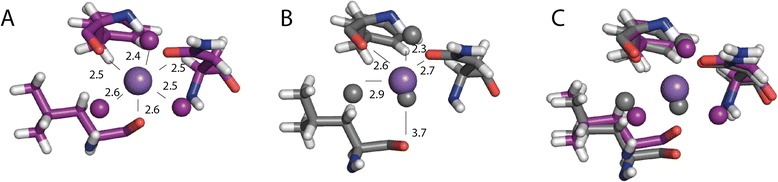
**Sodium ion binding site in Pil**_**Bac1**_ **ΔN. A**: Sodium ion binding site in chain B. The Na^+^ ion is coordinated by the backbone oxygens of Leu36 and Phe44, as well as the side chain oxygen of Asn38 and three water molecules. **B**: Homologous ion binding site in chain A with a superposed sodium ion from chain B. **C**: Superposition of the ion binding site in chain B, with the homologous residues in chain A. The carbonyl oxygen of Leu36 is moved further away from the sodium in chain A with respect to chain B increasing the distance to the Na^+^ ion to 3.7 Å. Molecules from chain A are shown in grey, molecules from chain B in purple. Distances in Å to the position of the sodium ion in chain B are shown in black.

In chain B clear density was observed, both in the anomalous map from the S-SAD data with a peak height of 6.5 σ (described in another manuscript) and in the 2mFo-DFc map from the high resolution data set, for an ion bound between the aβ-loop and the first β-strand. This ion was octahedrally coordinated by oxygen atoms (carbonyl oxygen of Leu36 and Phe44, delta oxygen of Asn38, 3 H_2_O molecules) with average coordination distances of 2.5 Å (Figure 7A). In agreement with these coordination properties, its anomalous scattering intensity and consistent with the presence of 100 mM NaCl in the buffer, we assigned this ion to a sodium ion (described in another manuscript).

Interestingly, this sodium ion was not observed in chain A due to a slight distortion of the ion binding site compared to chain B. The backbone of Leu36 is further away from the binding site increasing the distance from 2.6 to 3.7 Å (Figure 7B and C). Additionally, a third water molecule is missing to coordinate the ion; instead, another water molecule binds in the position where the sodium binds in chain B. Furthermore, features of positive density lie between the sodium ion binding site in chain A and a symmetry related molecule. This density is indicative of a bound molecule such as PEG which might have interfered with binding of the ion in this site.

So far, no functionally validated binding of any ions has been described for T4Ps. For pseudopilins the stabilization by calcium ions has been shown [68]. Whether the bound sodium ion in Pil_Bac1_ΔN fulfills a functional role or is a crystal artefact due to the 100 mM NaCl in the buffer, is not clear yet. Hypothetically, the Na^+^ ion could act as a regulator in filament assembly. In the extracellular space and periplasm, the sodium concentration is much higher than intracellularly and we find it possible that the pilin might bind a sodium ion in the periplasm.

### Modelling of a Pil_Bac1_ pilus

**Figure 8:**
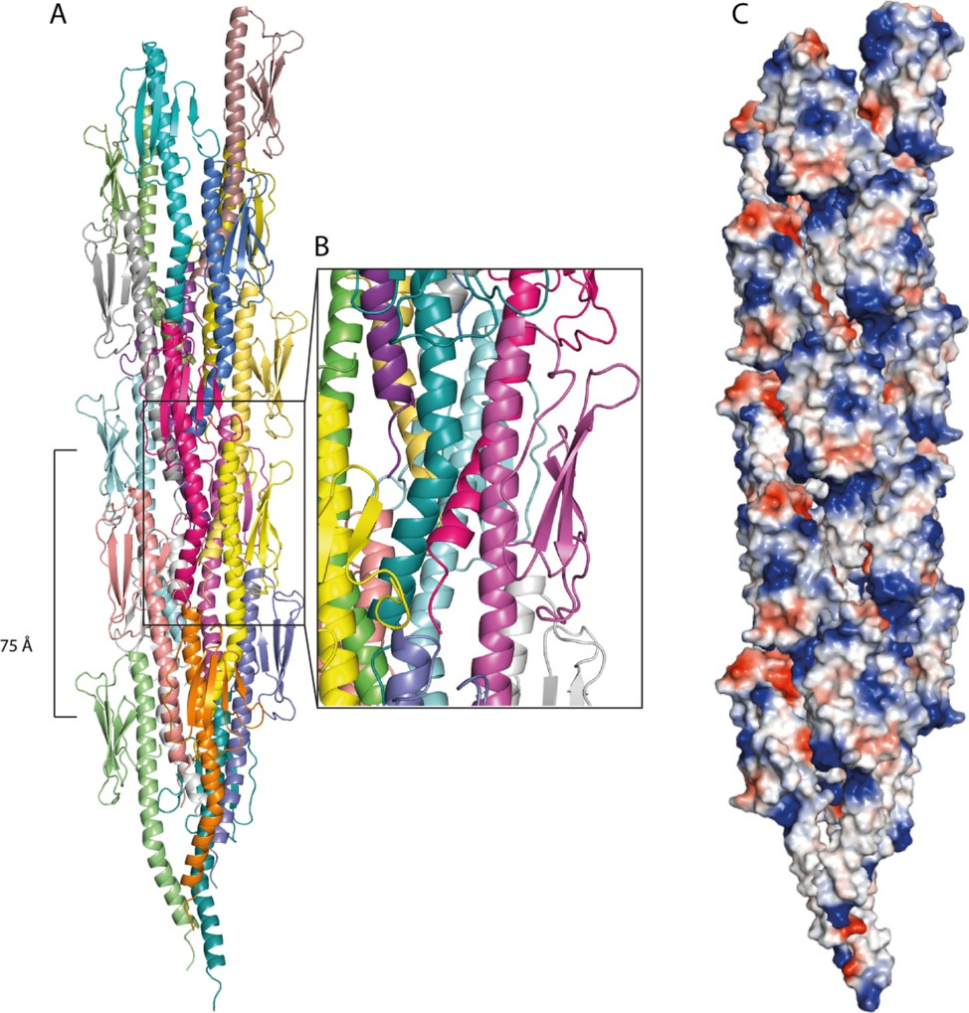
**Modelled pilus of Pil**_**Bac1**_ **molecules based on the** ***N. gonorrhoeae*** **pilus (2HIL.PDB).** Pil_Bac1_ molecules were superposed onto the PilE subunits from *N. gonorrhoeae* with PyMOL (PyMOL Molecular Graphics System, Version 1.5.0.4 Schrödinger, LLC). **A**: Overview. **B**: Magnified view of **A**. **C**: Surface representation of the electrostatic potential of the pilus.

To investigate the putative packing in a pilus, a model composed of Pil_Bac1_ subunits was generated (Figure 8). In order to do this, an atomic pilus model based on the EM structure of a PilE pilus and the crystal structures of PilE from *N. gonorrhoeae* were used as a template [34]. First, the structure of a Pil_Bac1_ΔN monomer was superimposed onto monomeric PilE and the missing N-terminal 28 residues were modelled resulting in a full-length model of Pil_Bac1_. This full-length chimera was then overlaid onto the individual subunits in the PilE pilus based on the modelled, very similar transmembrane part. No significant clashes between the subunits were introduced, only minor clashes between the N-terminal part of the helix of one monomer and the C-terminal part of the helix of the neighboring monomer (Figure 8B).

As for the *Neisseria* pilus, the α-helices formed the central core with the head domains facing outwards; however, in contrast to the Neisseria pilus, the packing seemed to be less dense due to the missing kink in the C-terminal part of the helix (kink 2) and due to a smaller head domain. The positively charged patch at the N-terminus from one pilin is located closely to the negatively charged patch of the neighbouring pilin stabilizing the interaction between pilins in the pilus filament. (Figure 8C). Overall, the Pil_Bac1_ pilus was a bit thinner than for *Neisseria*, because the head domain in Pil_Bac1_ is less bulky with no major grooves or protrusions on the surface.

This model for a Pil_Bac1_ pilus is in good agreement with the general observations that the D-region is involved in the interaction with other molecules [1,87] and should be solvent accessible. Still, it needs to be considered that this model is based on a model pilus from *N. gonorrhoeae* which in turn is based on the docking of the crystal structure of PilE into an EM envelope. Unlike PilE, Pil_Bac1_ does not contain a kink in the α-helix after the transmembrane part which will orient the head domain in a slightly different angle (kink2). This will necessarily affect the packing pattern in a pilus and inevitably lead to differences to the PilE pilus and therefore, this model has to be interpreted with some caution.

### Aromatic amino acids in Pil_Bac1_

**Figure 9:**
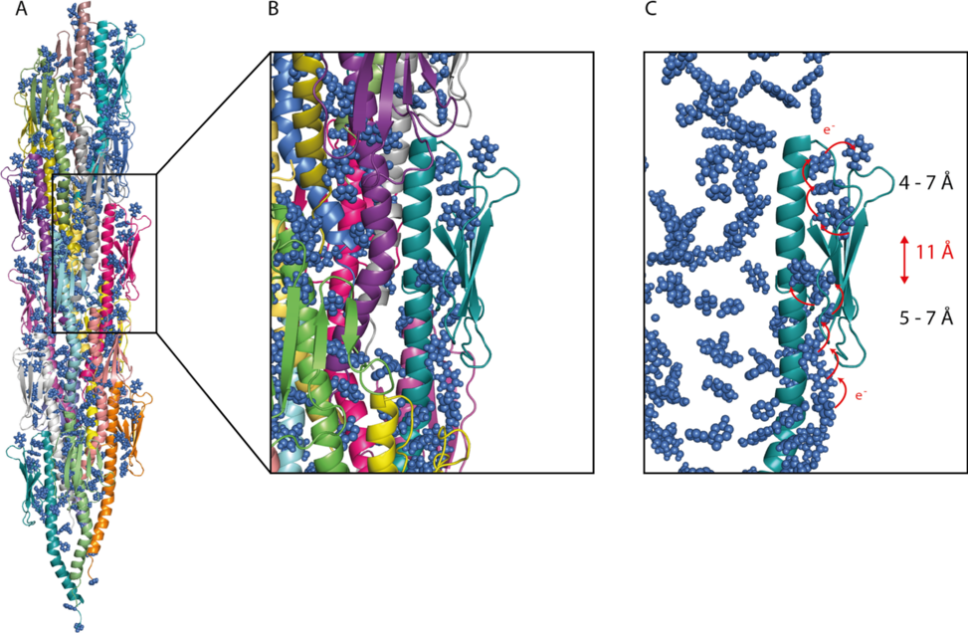
**Positioning of the aromatic residues in Pil**_**Bac1**_ **. A**: Overall alignment in the pilus. **B**, **C**: Magnified view on pilus subunits. The aromatic residues are shown as sphere representation in blue. Round, red arrows show the shortest distances between two neighboring aromatics. The distances between the individual aromatics are shown in Å on the side and the distance between two clusters is shown in Å in red. e^-^: electron.

Malvankar and co-workers have proposed that nanowires from *G. sulfurreducens* are conductive due to the close positioning of aromatic amino acids in PilA [27]. The NMR structure of PilA from *G. sulfurreducens* showed that the aromatic side chains were indeed closely spaced with a maximum distance of 15 Å [43]; yet, to the best of our knowledge, the maximum distance between aromatic groups that allows for electron transfer has not been defined so far. Similar to *G. sulfurreducens*, *S. oneidensis* forms conductive nanowires and, based on the overall similarity between these two organisms including metabolic pathways and the prevalence of multiheme cytochromes, a similar electron transfer mechanism is very likely. Pil_Bac1_ is the type IV pilin which is most closely related to PilA from *G. sulfurreducens* based on sequence comparisons (Additional file 2: Table S2). The full-length chimeric model of Pil_Bac1_ contained 14 aromatic residues including two phenylalanines and one tyrosine in the modelled transmembrane domain. In the modelled pilus of Pil_Bac1_ subunits, the aromatic side chains were evenly spaced throughout the whole structure, with some being closer to their neighbors than others (Figure 9A). A long chain of aromatic side chains wound along the modelled filament with two clusters on each subunit in which the aromatics are closely positioned to each other with distances between 4 to 7 Å. Yet, these two clusters are separated by a gap of 11 Å which can be defined as the maximum distance between two aromatics in the pilus model. This distance compares well to PilA from Geobacter; however, the arrangement of aromatic side chains in PilE from *N. gonorrhoeae* – which has not been shown to produce conductive nanowires yet – is similar with a maximum distance between individual aromatic side chains of around 13 Å (Additional file 8: Figure S4). This may argue against the hypothesis stating that conductivity is based on a specific alignment of aromatic side chains.

## Conclusions

In this study we described the high-resolution structure of the N-terminally truncated type IV pilin Pil_Bac1_ which exhibits the typical fold of type IV pilins with a long N-terminal α-helix packed against 4 antiparallel β-strands. Pil_Bac1_ was crystallized as a parallel dimer with a sodium ion bound to one of the monomers. Small-angle X-Ray scattering studies of the N-terminally truncated Pil_Bac1_ indicated that the protein exists as a monomer in solution, but further characterization of the full-length form and/or membrane bound form will be necessary to clarify the oligomeric state of Pil_Bac1_ in a cellular context. In contrast to most other T4aP head domains, Pil_Bac1_ displays a straight α-helix and a small head domain which leads to a less dense packing mode in a modelled pilus compared to other well-characterized pilins and possibly making room for interaction partners such as multiheme cytochromes.

## Methods

### Cloning, expression and purification

The DNA coding for residues 36 to 123 of Pil_Bac1_ from *S. oneidensis* with LIC overhangs at the 5’ and 3’ ends was amplified by Polymerase Chain Reaction (PCR) using genomic DNA from cell lysates as a template (forward primer: GACGACGACAAGATGGATTACGACATCCCCACTACT*GAGAATCTTTATTTTCAGGGC*AAGCAAGGCAGACGCTTCGATGCGC; reverse primer: GAGGAGAAGCCCGGTTTAATGGCTCCAACAATTTGTGGCGGGG). The PCR fragment obtained was then inserted into the vector pET-46 Ek/LIC by ligation independent cloning (LIC Kit, Novagen, USA). An N-terminal His tag and an Enterokinase (EK) site are part of pET-46 Ek/LIC vector and a sequence encoding a Tobacco Etch Virus (TEV) protease cleavage site (marked in italics above) was included in the forward primer from the PCR. The correct insert of the plasmid was verified by sequencing and it was transformed into *E. coli* BL 21 DE3 Origami cells (Novagen, USA).

6–12 L of LB medium (containing 100 μg/ml ampicillin and 50 μg/ml kanamycin) were inoculated with 60–120 ml of overnight culture and grown at 37°C at 120 rpm. When an OD600 between 0.6 and 0.8 was reached, expression was induced by the addition of Isopropyl-thiogalactoside (final concentration of 1 mM) for 18 hours at 20°C. Cells were harvested by centrifugation at 8,927.1 *g* for 20 minutes. The cell pellets were resuspended in 25 ml LB/ liter culture, flash-frozen in liquid nitrogen and stored at −20°C until use.

Cell pellets were resuspended in 3 ml lysis buffer (20 mM Tris–HCl pH 7.5, 500 mM KCl, 10% Glycerol) per gram wet cell pellet and opened by sonication on ice. Unopened cells and cell debris were spun down by centrifugation at approximately 235,000 *g* at 4°C for 2 h. Imidazole was added to the supernatant to a final concentration of 10 mM and loaded onto a 5 ml nickel-chelating column (GE Healthcare, USA) that had been pre-equilibrated in lysis buffer and 10 mM imidazole. After washing with lysis buffer with 10 mM imidazole, the protein was eluted on an ÄKTA Prime with a gradient from 25 – 500 mM imidazole over 10 column volumes at 1 ml/min and fractions containing Pil_Bac1_ were pooled. The N-terminal His-tag was cleaved off the protein by the TEV protease during dialysis against 1 l lysis buffer at 4°C for 12 h. The protein was then loaded onto a 5 ml nickel-chelating column (GE Healthcare, USA) and the flow-through containing the cleaved protein was collected and concentrated on a 5 molecular weight cut-off concentrator. Pil_Bac1_ was further purified by a size exclusion step on a Superdex 75 10/300 (GE Healthcare, USA) equilibrated in 20 mM Tris–HCl pH 7.5, 100 mM NaCl. Finally, Pil_Bac1_ was concentrated to 15 mg/ml and stored at −80°C.

### Synchrotron radiation circular dichroism

SRCD studies were performed at the CD1 beam line of the ASTRID synchrotron, Aarhus University, Denmark [88,89]. Light from the CD1 beam line passed through an MgF Rochon polarizer (B-Halle GmbH, Berlin) and alternating left and right circularly polarized light was produced using a photo-elastic modulator (Hinds, USA). The polarized light then passed through the sample and was detected by a photomultiplier tube (9406B, ETL, UK). SRCD spectra were taken from 280 nm to a minimum wavelength of 190 nm. To investigate the stability of the protein, spectra were recorded with increasing temperature from 7 to 81°C (5°C per step; 3 measurements at each temperature). After such a temperature scan, the sample was cooled down to 24°C and three final spectra were recorded after incubation at 24°C for 9 h.

All spectra were recorded at a concentration of 0.66 mg/ml, in 100 mM NaCl, 20 mM TRIS pH 7.5. Before and after each temperature scan, a spectrum of the buffer was recorded to check that no changes had occurred during the sample measurement (e.g. damage to the cell, changes in beam). The two buffer spectra were averaged and subtracted from the sample spectra using a spreadsheet operation.

Principal component analysis of the set of CD spectra recorded over the temperature range 7 to 81°C was performed using the Multibase 2013 (http://www.numericaldynamics.com/) add-in for excel to yield the component curves (Figure 2B) and corresponding coefficients for each temperature (Figure 2C). The resulting coefficients were each fitted by a sigmoidal function to find the inflection point and hence the melting temperature for that component.

### Thermofluor

A Thermofluor experiment was performed with Pil_Bac1_ΔN in 100 mM NaCl, 20 mM TRIS pH 7.5 (25 μM) and with 10xSYPRO Orange (Sigma Aldrich) using a Light Cycler 480 (Roche) and the option for protein melting dynamics. The temperature was increased from 20 to 85°C with 4.4°C per s and the fluorescence was measured with 20 acquisitions per time point. Plotting the fluorescence against the temperature yielded a sigmoidal curve. The inflection point of this curve was approximated as the melting temperature of Pil_Bac1_ΔN.

### X-Ray crystallography

#### Crystallization, data collection and processing

Pil_Bac1_ΔN was crystallized at a concentration of 15 mg/ml in a hanging drop set-up in a 2:1 ratio with the reservoir (26% PEG 8,000, 0.15 M (NH_4_)_2_SO_4_ and 0.1 M CHES pH 8.6) at 19°C. Rod shaped crystals appeared after a few days and were stored in liquid nitrogen without the addition of more cryoprotectant.

A high resolution data set was collected from crystals at beamline BL14.2, Bessy II, Berlin, [90] at 0.98 Å with 0.5 s of exposure time (Table 1). All data were processed and merged with the XDS software [59]. The Wilson B-factor was determined by the program AIMLESS [91]. The Matthews coefficient [92] and the solvent content were derived by the program XTRIAGE from the Phenix suite [60]. See also Table 1 for data collection and processing statistics.

#### Structure determination and analysis

The S-SAD structure of Pil_Bac1_ΔN (PDB accession code 4US7) was used as a search model for molecular replacement using the program PHASER [93] from the Phenix suite. The model was refined with Phenix.Refine [60] with the options for xyz coordinates, TLS, individual B-factors, optimizing X-Ray stereochemical weights and ADP weights with iterative model building in Coot [61]. In the last steps, the model was refined including riding hydrogen atoms. The model was validated with the program MOLPROBITY [94] and deposited in the protein data bank with the accession code 4D40.

The electrostatics for the monomer were calculated using the PDB2PQR (version 1.8) server at http://nbcr-222.ucsd.edu/pdb2pqr_1.9.0/ with standard parameters and force field = PARSE. Files from PDB2PQR were used with the APBS [95] plugin in PyMOL 1.7.4 to generate images, coloring a range of +/− 5 kT/e by potential on the solvent accessible surface. The electrostatics for the pilus model was calculated using the built-in feature of PyMOL.

The structure of Pil_Bac1_ΔN was superposed onto PilE from *N. gonorrhoeae* (PDB accession code 2HI2) [34] based on the C_α_s of the α-helix and the first two β-strands and the first 28 residues of PilE were then added onto Pil_Bac1_ΔN using PyMOL (PyMOL Molecular Graphics System, Version 1.5.0.4 Schrödinger, LLC). Residues 9 (V) and 23 to 28 (AYQDYT) were then mutated into the corresponding residues in Pil_Bac1_ (A; SFNFYL) and the modelled helix was subjected to energy minimization using torsion angle and Ramachandran constraints in COOT [61]. The minimized chimera structure was superposed on the first 28 residues of the subunits in the Neisseria PilE pilus model (PDB accession code 2HIL) [34] in order to generate a Pil_Bac1_ΔN pilus model.

### Small-angle X-Ray scattering

#### Data collection

The data collection parameters are given in Additional file 6: Table S4. Before and after a scattering profile of the protein was recorded, SAXS data of the buffer were collected. The data for the buffer were merged and subtracted from the protein (in buffer) scattering curve to yield the protein scattering curve.

#### Data processing and model building

The scattering data were processed with programs from the ATSAS package (PRIMUS [96], DAMMIF [81] DAMAVER [97]). The radius of gyration and the maximum diameter of the protein were calculated with the PRIMUS and GNOM programs. 12 *ab initio* models consisting of dummy atoms were made with DAMMIF. The models from DAMMIF were evaluated with DAMAVER and for all models, the normalized spatial discrepancy (NSD) deviated no more than two standard variations from the mean value, thus no models were excluded [97]. Envelopes were made based on the DAMMIF model displaying the lowest NSD to the remaining models using the SITUS package and the pdb2vol program using default settings [85]. The X-Ray structure of Pil_Bac1_ΔN was docked into the envelope obtained from pdb2vol using the program SUPCOMB [82] and the model was visualized in UCSF Chimera [83]. To compare the SAXS model to the crystal structure, a theoretical scattering curve was generated based on the crystal structure using CRYSOL [84].

## Additional files

Additional file 1: Table S1. Structures of T4Ps and pseudopilins. “b” indicates a β-sheet, “a” an α-helix [98-103]. Additional file 2: Table S2. Sequence identities between T4Ps from *S. oneidensis* (Pil_Bac1_So, PilESo, PilASo, MshASo, MshBSo, Pil_Bac2_So, Pil_Bac3_So, Pil_Bac4_So, PilVSo, PilXSo) with PilA from *G. sulfurreducens*. The alignment was done using the program MUSCLE [53]. Additional file 3: Figure S1. Sequence alignment of T4Ps from *S. oneidensis* (Pil_Bac1_So, PilESo, PilASo, MshASo, MshBSo, Pil_Bac2_So, Pil_Bac3_So, Pil_Bac4_So, PilVSo, PilXSo) with PilA from *G. sulfurreducens*. The alignment was done using the program MUSCLE [53]. Additional file 4: Figure S2. Positive density at the interface to the solvent channels. A: Overview over the unit cell. B: Positive density at residues 3 and 5 in chain A. The 2Fo-Fc map was contoured at 1.5σ, Fo-Fc map at 3σ. Additional file 5: Table S3. Identification of homologous proteins to Pil_Bac1_∆N by the DALI server [65]. The R.M.S.D. value between the structures obtained from DALI based on C_αs_ is given. Additionally, the number of residues in the structure is given. The three most homologous T4P structures and the most homologous pseudopilin structure are highlighted in bold. Additional file 6: Table S4. SAXS data collection parameters and data processing statistics. The dry volume was calculated by an online server based on considerations from Harpaz et al. [104]. The molecular mass was determined by Porod volume analysis with the program AUTOPOROD [105]. Additional file 7: Figure S3. SAXS data. A: Original SAXS curves. B: SAXS curve for 16 mg/ml. C: Guinier plot for 16 mg/ml. D: Porod plot for 16 mg/ml. E: Kratky plot for 16 mg/ml. F: Pair-distance distribution plot p(r). s: momentum transfer, I: scattering intensity. Additional file 8: Figure S4. Conservation of aromatic amino acids in Pil_Bac1_ from *S. oneidensis* (blue), PilA from *G. sulfurreducens* (yellow) and PilE from *N. gonnorhoeae* (red). A: Cartoon presentation of all three pilins. B: Cartoon presentation of PilA from *G. sulfurreducens* and the aromatics in all three pilins shown as sticks.

## Abbreviations

EET: Extracellular Electron Transport
EM: Electron Microscopy
NMR: Nuclear Magnetic Resonance
ORF: Open Reading Frame
PCA: Principle Component Analysis
PISA: Proteins, Interfaces, Structures and Assemblies
SAXS: Small-Angle X-Ray Scattering
SRCD: Synchrotron Radiation Circular Dichroism
S-SAD: Sulfur-Single-wavelength Anomalous Diffraction
T4P: Type IV Pilin
