# Supplementary material for: High-resolution structure of a type IV pilin from the metal-reducing bacterium Shewanella oneidensis
Source: BMC Struct Biol. 2015 Feb 27;15:4. doi: 10.1186/s12900-015-0031-7 (PMC4376143; doi:10.1186/s12900-015-0031-7)
Supplement: Additional file 1: Table S1. — Structures of T4Ps and pseudopilins. “b” indicates a β-sheet, “a” an α-helix [98-103]. [file 12900_2015_31_MOESM1_ESM.docx]

Table S1: Structures of T4Ps and pseudopilins. “b” indicates a β-sheet, “a” an α-helix.

|  | Protein | Organism | Form | PDB Code | Uniprot Code | Disulfide bridge | Kink2 |  | Reference |
| --- | --- | --- | --- | --- | --- | --- | --- | --- | --- |
|  | PilE1 | *N. gonorrhoeae* | Full-Length | 2PIL | P02974 | b4-loop after b6 | Yes |  | [[80](#_ENREF_98)] |
|  | PilE1 | *N. gonorrhoeae* | Full-Length | 2HI2 | P02974 | b4-loop after b6 | Yes |  | [[34](#_ENREF_99)] |
|  | PilA | *P.aeruginosa* PAK | Full-Length | 1OQW | P02973 | b4-last loop | Yes |  | [[32](#_ENREF_100)] |
|  | FimA | *D. nodosus* | Full-Length | 3SOK | P02795 | b3-ab-loop | Yes |  | [77] |
|  | PilA | *G. sulfurreducens* | Full-Length | 2M7G | GSU1496 | - | No |  | [[43](#_ENREF_43)] |
|  | PilA | *P.aeruginosa* K122-4 | N-terminally truncated | 3JYZ | Q8KQ36 | b4-last loop with helix | Yes |  | [[44](#_ENREF_102)] |
|  | PilA | *P.aeruginosa*  K122-4 | N-terminally truncated | 1QVE | P17838 | b4-last loop with helix | Yes |  | [[74](#_ENREF_103)] |
|  | PilA | *P.aeruginosa* PAK | N-terminally truncated | 1X6Z | P02973 | b4-last loop | Yes |  | [[67](#_ENREF_104)] |
|  | PilA | *P.aeruginosa* PAK | N-terminally truncated | 1DZO | P02973 | b4-last loop | Yes |  | [98] |
|  | Pilin protein | *F. tularensis* | N-terminally truncated | 3SOJ | Q5NGF6 | b4-last loop with helix | Yes |  | [[77](#_ENREF_101)] |
|  | CompA | *N. mengitidis* | N-terminally truncated | 2M3K | C9X2N5 | b4-last loop | No |  | [[4](#_ENREF_4)] |
|  | TcpA | *V. cholerae* | N-terminally truncated | 3HRV | Q60153 | a3-last loop/last helix (T4bP) | No |  | [[76](#_ENREF_76)] |
|  | PilS | *S. typhi* | N-terminally truncated | 3FHU | Q8Z1L1 | a3-a4 (T4bP) | No |  | [[99](#_ENREF_106)] |
|  | CofA | *Enterotoxic E. coli* | N-terminally truncated | 3S0T | Q59393 | a4-loopb3-a5 (T4bP) | Yes |  | [[78](#_ENREF_78)] |
|  | PilA_4 | *T. thermophilus* | N-terminally truncated | 4BHR | Q5SIZ3 | b4-last loop | No |  | [[64](#_ENREF_64)] |
|  | PilX | *N. meningitidis* | N-terminally truncated | 2OPE | A1IRD3 | b3-b4 | No |  | [[100](#_ENREF_107)] |
| Pseudopilins | GpsI:GpsJ | *V. cholerae* | N-terminally truncated | 2RET | Q7MPZ0; Q7MPZ1 | - | No |  | [[52](#_ENREF_52)] |
|  | GspH | *E. coli* | N-terminally truncated | 2KNQ | P41443 | - | No |  | To be published |
|  | Major pseudopiloin | *V. cholerae* | N-terminally truncated | 3FU1 | P45773 | - | No |  | [[68](#_ENREF_68)] |
|  | PulG | *V. vulnificus* | N-terminally truncated | 3GN9 | Q8DDT3 | - | Ni |  | [[68](#_ENREF_68)] |
|  | PulG | *E. coli (EHEC)* | N-terminally truncated | 3G20 | Q7BSV8 | - | No |  | [[68](#_ENREF_68)] |
|  | XcpT | *P. aeruginosa* | N-terminally truncated | 2KEP | Q00154 | - | No |  | [[101](#_ENREF_108)] |
|  | EpsH | *V. cholerae* | N-terminally truncated | 2QV8 | P45774 | - | No |  | [[75](#_ENREF_75)] |
|  | XcpW | *P. aeruginosa* | N-terminally truncated | 3NJE | Q00517 | - | No |  | [[102](#_ENREF_109)] |
|  | PulG | *K.oxytoca* | N-terminally truncated | 1T92 | P15746 | - | No |  | [[51](#_ENREF_51)] |
|  | GpsK:GpsI:GpsJ | *E. coli* | N-terminally truncated | 3CI0 | Q8VPC3 | - | No |  | [[103](#_ENREF_110)] |
