# Supplementary material for: High-resolution structure of a type IV pilin from the metal-reducing bacterium Shewanella oneidensis
Source: BMC Struct Biol. 2015 Feb 27;15:4. doi: 10.1186/s12900-015-0031-7 (PMC4376143; doi:10.1186/s12900-015-0031-7)
Supplement: Additional file 2: Table S2. — Sequence identities between T4Ps from S. oneidensis (PilBac1So, PilESo, PilASo, MshASo, MshBSo, PilBac2So, PilBac3So, PilBac4So, PilVSo, PilXSo) with PilA from G. sulfurreducens. The alignment was done using the program MUSCLE [53]. [file 12900_2015_31_MOESM2_ESM.docx]

Supplementary Table S2

|  | PilAGs | Pil_Bac1_So | PilESo | PilASo | MshASo | MshBSo | Pil_Bac2_So | Pil_Bac4_So | PilVSo | PilXSo | Pil_Bac3_So |
| --- | --- | --- | --- | --- | --- | --- | --- | --- | --- | --- | --- |
| PilAGs | 100 | 48 | 39 | 38 | 30 | 30 | 28 | 17 | 14 | 13 | 8 |
| Pil_Bac1_So | 48 | 100 | 44 | 36 | 33 | 27 | 30 | 22 | 21 | 12 | 10 |
| PilESo | 39 | 44 | 100 | 41 | 28 | 23 | 30 | 15 | 14 | 15 | 13 |
| PilASo | 38 | 36 | 41 | 100 | 32 | 35 | 38 | 18 | 13 | 20 | 12 |
| MshASo | 30 | 33 | 28 | 32 | 100 | 44 | 32 | 22 | 16 | 10 | 13 |
| MshBSo | 30 | 27 | 23 | 35 | 44 | 100 | 22 | 25 | 18 | 13 | 13 |
| Pil_Bac2_So | 28 | 30 | 30 | 38 | 32 | 22 | 100 | 18 | 20 | 16 | 17 |
| Pil_Bac4_So | 17 | 22 | 15 | 18 | 22 | 25 | 18 | 100 | 24 | 13 | 12 |
| PilVSo | 14 | 21 | 14 | 13 | 16 | 18 | 20 | 24 | 100 | 16 | 27 |
| PilXSo | 13 | 12 | 15 | 20 | 10 | 13 | 16 | 13 | 16 | 100 | 12 |
| Pil_Bac3_So | 8 | 10 | 13 | 12 | 13 | 13 | 17 | 12 | 27 | 12 | 100 |
