# Supplementary figures and images for: High-resolution structure of a type IV pilin from the metal-reducing bacterium Shewanella oneidensis
Source: BMC Struct Biol. 2015 Feb 27;15:4. doi: 10.1186/s12900-015-0031-7 (PMC4376143; doi:10.1186/s12900-015-0031-7)

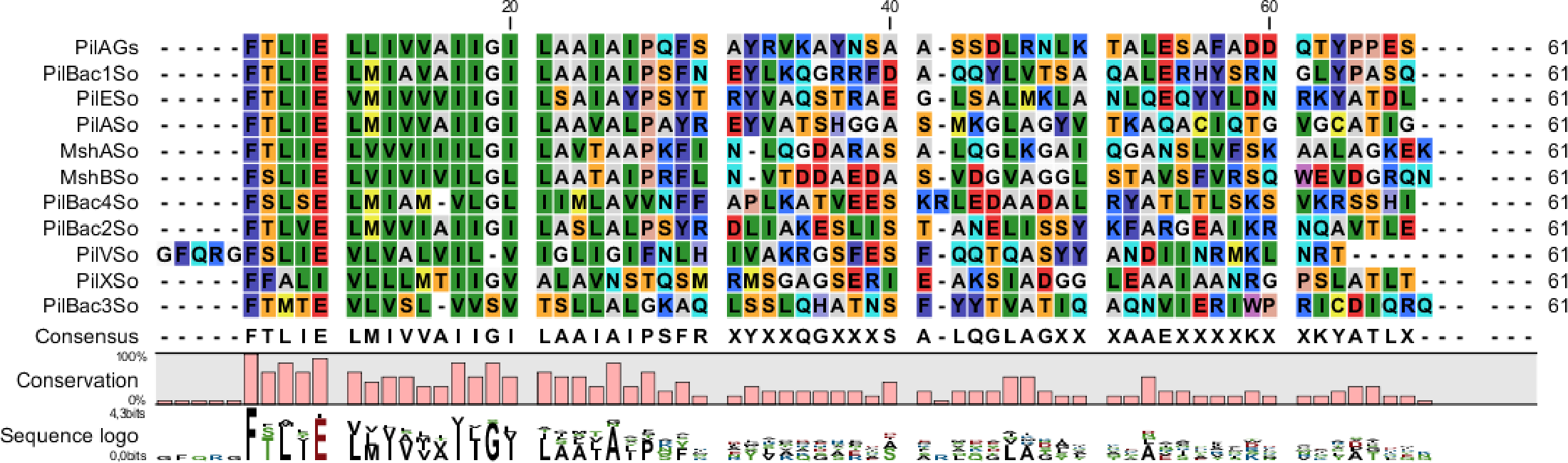

Supplement: Additional file 3: Figure S1. — Sequence alignment of T4Ps from S. oneidensis (PilBac1So, PilESo, PilASo, MshASo, MshBSo, PilBac2So, PilBac3So, PilBac4So, PilVSo, PilXSo) with PilA from G. sulfurreducens. The alignment was done using the program MUSCLE [53]. [file 12900_2015_31_MOESM3_ESM.tiff]

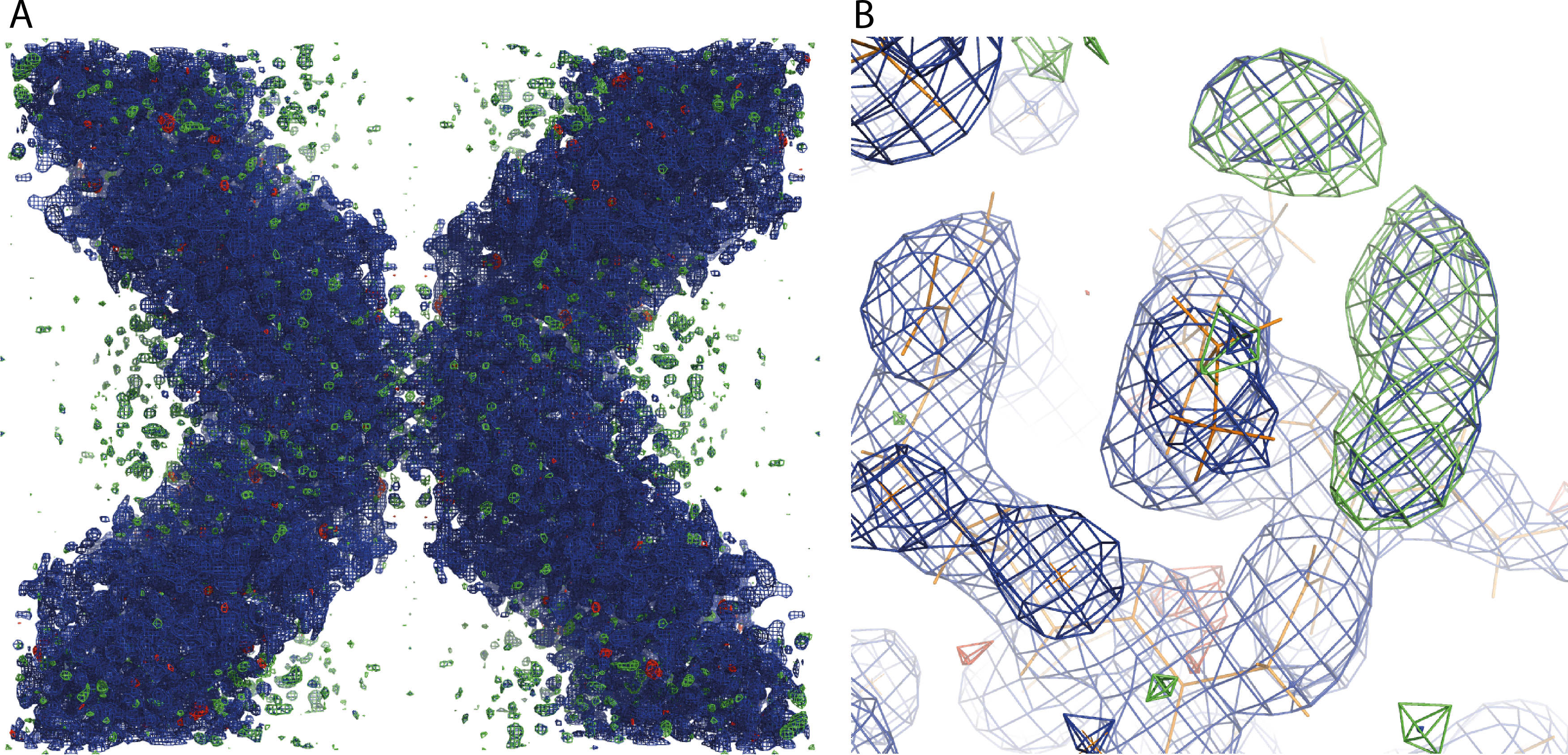

Supplement: Additional file 4: Figure S2. — Positive density at the interface to the solvent channels. A: Overview over the unit cell. B: Positive density at residues 3 and 5 in chain A. The 2Fo-Fc map was contoured at 1.5σ, Fo-Fc map at 3σ. [file 12900_2015_31_MOESM4_ESM.png]

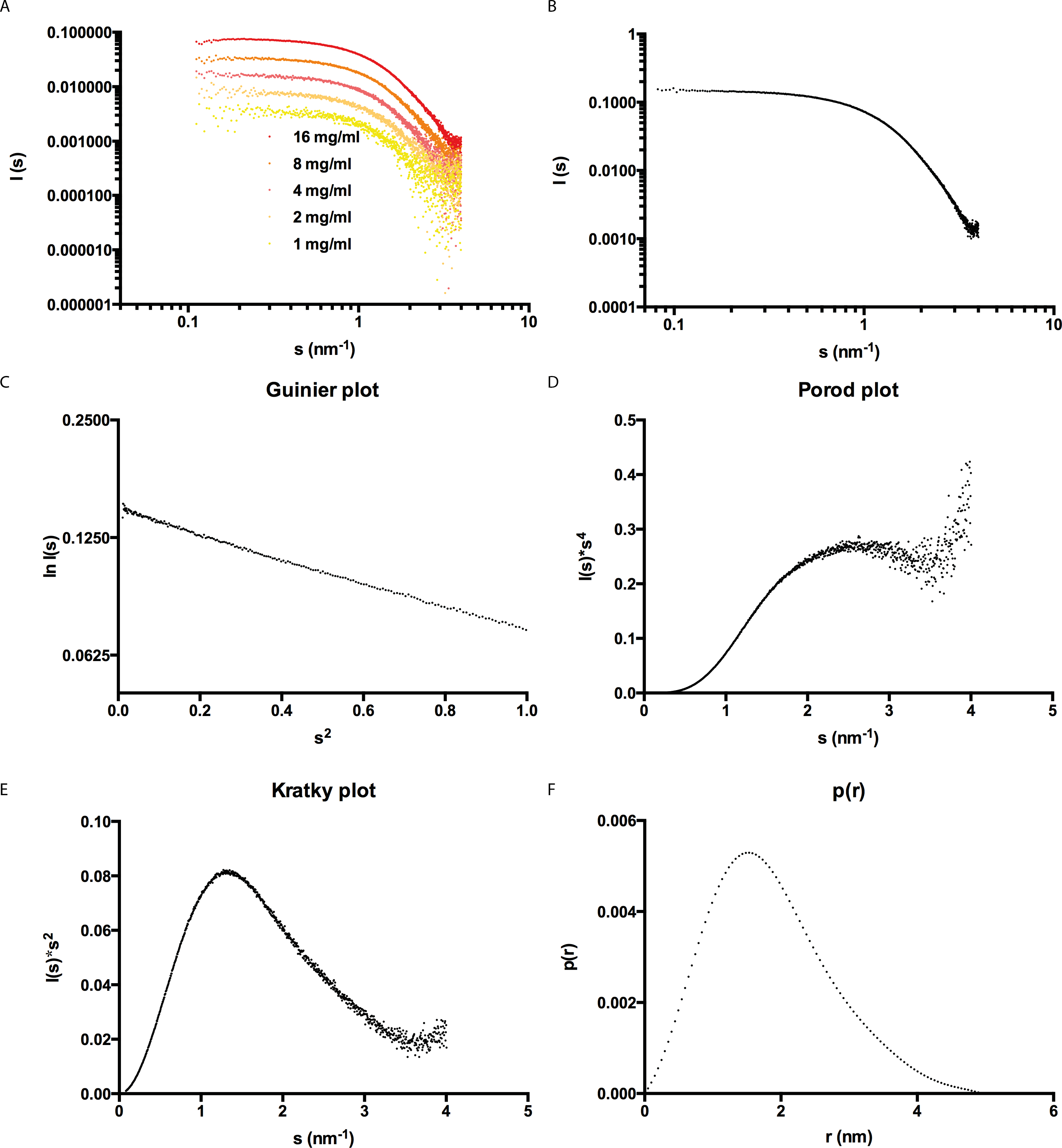

Supplement: Additional file 7: Figure S3. — SAXS data. A: Original SAXS curves. B: SAXS curve for 16 mg/ml. C: Guinier plot for 16 mg/ml. D: Porod plot for 16 mg/ml. E: Kratky plot for 16 mg/ml. F: Pair-distance distribution plot p(r). s: momentum transfer, I: scattering intensity. [file 12900_2015_31_MOESM7_ESM.png]

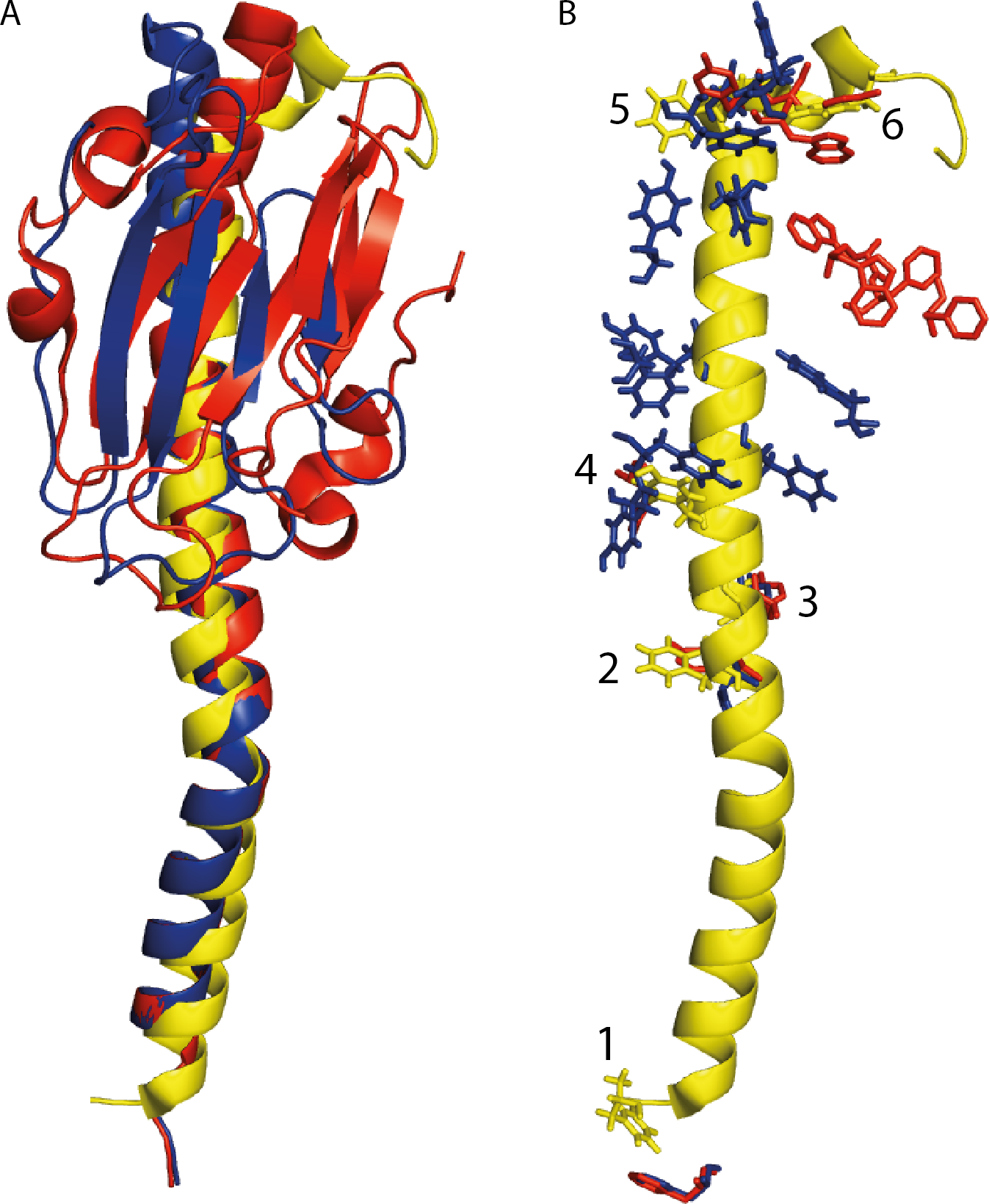

Supplement: Additional file 8: Figure S4. — Conservation of aromatic amino acids in PilBac1 from S. oneidensis (blue), PilA from G. sulfurreducens (yellow) and PilE from N. gonnorhoeae (red). A: Cartoon presentation of all three pilins. B: Cartoon presentation of PilA from G. sulfurreducens and the aromatics in all three pilins shown as sticks. [file 12900_2015_31_MOESM8_ESM.png]
