# Supplementary material for: High-resolution structure of a type IV pilin from the metal-reducing bacterium Shewanella oneidensis
Source: BMC Struct Biol. 2015 Feb 27;15:4. doi: 10.1186/s12900-015-0031-7 (PMC4376143; doi:10.1186/s12900-015-0031-7)
Supplement: Additional file 5: Table S3. — Identification of homologous proteins to PilBac1∆N by the DALI server [65]. The R.M.S.D. value between the structures obtained from DALI based on Cαs is given. Additionally, the number of residues in the structure is given. The three most homologous T4P structures and the most homologous pseudopilin structure are highlighted in bold. [file 12900_2015_31_MOESM5_ESM.docx]

Supplementary Table S3

|  | PDB Code | Chain | Name | Organism | R.M.S.D. | Score | Residues in structure |
| --- | --- | --- | --- | --- | --- | --- | --- |
|  |  | B | Pil_Bac1_ | *S. oneidenis* |  |  | 89 |
| 1 | **4BHR** | B | PilA_4 | *T. thermophilus* | 2.1 | 8.7 | 84 |
| 2 | **2OPD** | A | PilX | *N. meningitides* | 2.7 | 8.3 | 121 |
| 3 | 2OPE | D | PilX | *N. meningitides* | 2.6 | 8.2 | 121 |
| 4 | **1X6Z** | A | PAK pilin | *P. aeruginosa* | 2.2 | 8.1 | 120 |
| 5 | 1X6Q | A | PAK pilin | *P. aeruginosa* | 2.2 | 8.1 | 120 |
| 6 | 2OPD | B | PilX | *N. meningitides* | 2.5 | 8.0 | 121 |
| 7 | 2OPE | C | PilX | *N. meningitides* | 2.9 | 8.0 | 121 |
| 8 | 1X6R | A | PAK pilin | *P. aeruginosa* | 2.5 | 7.9 | 120 |
| 9 | 1DZO | A | PAK pilin | *P. aeruginosa* | 2.2 | 7.9 | 120 |
| 10 | 1X6Y | A | PAK pilin | *P. aeruginosa* | 2.5 | 7.9 | 120 |
| 11 | 1X6P | A | PAK pilin | *P. aeruginosa* | 2.5 | 7.9 | 120 |
| 12 | 2PY0 | A | Cs1 pilin chimera | *P. aeruginosa* | 2.5 | 7.9 | 120 |
| 13 | 2OPE | A | PilX | *N. meningitides* | 3.2 | 7.9 | 121 |
| 14 | 2OPE | B | PilX | *N. meningitides* | 3.3 | 7.9 | 121 |
| 15 | 4BHR | A | PilA_4 | *T. thermophilus* | 2.2 | 7.9 | 84 |
| 16 | 1RG0 | A | K122-4 pilin | *P. aeruginosa* | 3.1 | 7.7 | 126 |
| 17 | 1RG0 | B | K122-4 pilin | *P. aeruginosa* | 3.1 | 7.5 | 126 |
| 18 | 3JYZ | A | T4P | *P. aeruginosa* | 2.7 | 7.4 | 150 |
| 19 | 3SOJ | B | PilE | *F. tularensis* | 2.4 | 7.4 | 114 |
| 20 | 3SOJ | A | PilE | *F. tularensis* | 2.5 | 7.4 | 114 |
| 21 | 1OQW | A | PAK pilin | *P. aeruginosa* | 2.6 | 7.1 | 144 |
| 22 | 1OQW | B | PAK pili | *P. aeruginosa* | 2.6 | 7.1 | 144 |
| 23 | 3JZZ | A | T4P | *P. aeruginosa* | 2.7 | 7.0 | 148 |
| 24 | 1AY2 | A | T4P | *P. aeruginosa* | 2.4 | 7.0 | 158 |
| 25 | **4LW9** | J | EpsG | *V. cholerae* | 2.5 | 6.9 | 119 |
| 26 | 2HI2 | A | PilE | *N. gonorrhoeae* | 2.4 | 6.8 | 158 |
| 27 | 4LW9 | A | EpsG | *V. cholerae* | 2.6 | 6.8 | 119 |
| 28 | 4LW9 | L | EpsG | *V. cholerae* | 2.6 | 6.8 | 119 |
| 29 | 4LW9 | E | EpsG | *V. cholerae* | 2.6 | 6.8 | 119 |
| 30 | 4LW9 | D | EpsG | *V. cholerae* | 2.5 | 6.8 | 119 |
| 31 | 4LW9 | B | EpsG | *V. cholerae* | 2.6 | 6.8 | 119 |
| 32 | 4LW9 | K | EpsG | *V. cholerae* | 2.6 | 6.8 | 119 |
| 33 | 4LW9 | Q | EpsG | *V. cholerae* | 2.6 | 6.8 | 119 |
| 34 | 4LW9 | R | EpsG | *V. cholerae* | 2.5 | 6.8 | 119 |
| 35 | 4LW9 | C | EpsG | *V. cholerae* | 2.6 | 6.8 | 119 |
| 36 | 4LW9 | I | EpsG | *V. cholerae* | 2.6 | 6.8 | 119 |
| 37 | 3GN9 | B | EpsG | *V. vulnificus* | 2.4 | 6.8 | 112 |
| 38 | 3GN9 | A | EpsG | *V. vulnificus* | 2.5 | 6.8 | 112 |
| 39 | 3GN9 | C | EpsG | *V. vulnificus* | 2.3 | 6.8 | 112 |
| 40 | 3FU1 | B | EpsG | *V. cholerae* | 2.3 | 6.6 | 115 |
| 41 | 2HIL | P | PilE | *N. gonorrhoeae* | 2.5 | 6.5 | 158 |
| 42 | 2HIL | E | PilE | *N. gonorrhoeae* | 2.5 | 6.5 | 158 |
| 43 | 2HIL | N | PilE | *N. gonorrhoeae* | 2.5 | 6.5 | 158 |
| 44 | 2HIL | A | PilE | *N. gonorrhoeae* | 2.5 | 6.5 | 158 |
| 45 | 3FU1 | A | EpsG | *V. cholerae* | 2.3 | 6.5 | 115 |
| 46 | 2HIL | L | PilE | *N. gonorrhoeae* | 2.5 | 6.4 | 158 |
| 47 | 2HIL | J | PilE | *N. gonorrhoeae* | 2.5 | 6.4 | 158 |
| 48 | 2HIL | D | PilE | *N. gonorrhoeae* | 2.5 | 6.4 | 158 |
| 49 | 2HIL | M | PilE | *N. gonorrhoeae* | 2.5 | 6.4 | 158 |
| 50 | 2HIL | G | PilE | *N. gonorrhoeae* | 2.5 | 6.4 | 158 |
