# Supplementary material for: High-resolution structure of a type IV pilin from the metal-reducing bacterium Shewanella oneidensis
Source: BMC Struct Biol. 2015 Feb 27;15:4. doi: 10.1186/s12900-015-0031-7 (PMC4376143; doi:10.1186/s12900-015-0031-7)
Supplement: Additional file 6: Table S4. — SAXS data collection parameters and data processing statistics. The dry volume was calculated by an online server based on considerations from Harpaz et al. [104]. The molecular mass was determined by Porod volume analysis with the program AUTOPOROD [105]. [file 12900_2015_31_MOESM6_ESM.docx]

Supplementary Table S4

| Data collection parameters |  |
| --- | --- |
| Instrument | Maxlab IV, I911-4 |
| Beam geometry (mm^2^) | 0.3*0.3 |
| Wavelenth (Å) | 0.91 |
| Q range (Å^-1^) | 0.01 – 0.43 |
| Exposure time (min) | 2 |
| Concentration range (mg ml-1) | 16, 8, 4, 2,1 0.5 |
| Temperature (K) | 283 |
| Detector_name | Pilatus_1M |
| Detector binning | 1 |
|  |  |
| Structural parameters reported for 16 mg ml^-1^ |  |
| I_0_ (cm^-1^) from P(r) | 0.14 |
| R_g_ (Å) from P(r) | 15 |
| I_0_ (cm^-1^) from Guinier | 0.14 |
| R_g_ (Å) from Guinier | 15 |
| D_max_ (Å) | 49 |
| Porod volume estimate (Å^3^) | 9,008 |
| Dry volume calculated from sequence (Å^3^) | 11,911 |
| Molecular Mass Determination (reported per 16 mg ml^-1^)^1^ | 6.0 |
